# Live Effects of Anodal and Cathodal Transcranial Direct Current Stimulation on Brain Metabolism in a Patient with Typical Hemorrhagic Stroke: A Case Study

**DOI:** 10.3390/brainsci15060594

**Published:** 2025-06-01

**Authors:** Giuseppe Reale, Augusto Fusco, Fabrizio Cocciolillo, Vincenza Amoruso, Davide Glorioso, Maria Caputo, Maria Lucia Calcagni, Luca Padua

**Affiliations:** 1 UOC Neuroriabilitazione ad Alta Intensità, Dipartimento di Neuroscienze, Organi di Senso e Torace, Fondazione Policlinico Universitario Agostino Gemelli IRCCS, 00168 Rome, Italy; giuseppe.reale@policlinicogemelli.it (G.R.); luca.padua@unicatt.it (L.P.); 2 UOS Neuroriabilitazione ad Alta Specialità, Dipartimento di Neuroscienze, Organi di Senso e Torace, Fondazione Policlinico Universitario Agostino Gemelli IRCCS, 00168 Rome, Italy; 3 Unità di Medicina Nucleare, Dipartimento di Diagnostica per Immagini e Radioterapia Oncologica, Fondazione Policlinico Universitario Agostino Gemelli IRCCS, 00168 Rome, Italy; fabrizio.cocciolillo@policlinicogemelli.it (F.C.); marialucia.calcagni@unicatt.it (M.L.C.); 4 Medicina Fisica e Riabilitazione, Dipartimento di Scienze Geriatriche e Ortopediche, Università Cattolica del Sacro Cuore, 00168 Rome, Italy; vincenza.amoruso90@gmail.com (V.A.); davideglorioso91@gmail.com (D.G.); 5 Dipartimento di Scienze Biomediche, Metaboliche e Neurali, Università di Modena e Reggio Emilia, 41121 Modena, Italy; caputomaria03@gmail.com; 6 Istituto di Medicina Nucleare, Università Cattolica del Sacro Cuore, 00168 Rome, Italy

**Keywords:** transcranial direct current stimulation, positron emission tomography computed tomography, stroke, fluorodeoxyglucose F18, interhemispheric inhibition

## Abstract

In this study, we aimed to assess the effects of transcranial direct current stimulation (tDCS) stimulation on brain metabolism in a patient with typical hemorrhagic stroke in a subacute phase. The patient was evaluated with ^18^F-FDG PET (18F-fluoro-2-deoxy-D-glucose positron emission tomography) during tDCS brain stimulation at 6, 8, and 10 weeks from the event. The patient underwent the following protocol: baseline cerebral ^18^F-FDG-PET (T0); cerebral ^18^F-FDG-PET during anodal-tDCS on the affected hemisphere (T1); and cerebral ^18^F-FDG-PET during cathodal-tDCS on the unaffected hemisphere (T2). Baseline PET examination revealed marked hypometabolism of the right nucleo-capsular hemorrhagic lesion; at T1, an increase in brain metabolism was shown in the stimulated hemisphere and unexpectedly in the non-stimulated hemisphere; at T2, a reduction in metabolism was documented in the hemisphere ipsilateral to the inhibiting current applied by tDCS. The use of PET may provide new insights into the effects of tDCS on brain metabolism, providing in vivo information about the plasticity mechanisms of the injured brain. Further studies, using a combination of PET and tDCS, are necessary to further clarify the mechanisms of action of this stimulation technique to the clinical and functional outcomes.

## 1. Introduction

A stroke analysis based on the Global Burden of Disease study revealed that stroke represents the second most prevalent cause of death in Western countries and the leading cause of disability among adults [1]. As the population continues to age and survival rates improve, the prevalence of stroke is expected to rise. Even if the clinical outcomes of stroke vary, lasting impairments may occur, significantly impacting an individual’s physical, emotional, and social well-being [2]. Hence, rehabilitation is essential to reduce the risk of long-term disability, with interventions customized to meet the specific needs of each patient.

Among the different techniques available, transcranial direct current stimulation (tDCS) is considered a valuable tool for the rehabilitation of stroke, with its efficacy being demonstrated in the promotion of cognitive and motor recovery. This non-invasive brain stimulation technique applies a mild direct electrical current (1–2 mA) to the scalp to enhance or diminish neuronal excitability [3,4]. Several studies have demonstrated the improvement in motor and cognitive functions in both healthy subjects and individuals affected by various neurological disorders [5,6]. In patients with stroke, two main approaches have been proposed: the stimulation of the affected hemisphere with anodal-tDCS to increase its excitability and/or the inhibition of the unaffected hemisphere with cathodal-tDCS to reduce interhemispheric inhibition [7].

These protocols were designed based on the “interhemispheric rivalry model” to restore balance between the cerebral hemispheres [7]. According to the aforementioned model, the hemispheres are hypothesized to be functionally connected through transcallosal pathways, thereby maintaining equilibrium via mutual inhibition. However, following a brain injury, this balance is disrupted. The reciprocal inhibitory modulation between hemispheres becomes asymmetrical, with the uninjured hemisphere exerting excessive pathological inhibition on the damaged one, further reducing its functionality [7,8]. More recently, it has been suggested in the literature that this model may represent an oversimplified view of the complex and dynamic interactions in the brain during stroke recovery. Several studies have demonstrated that there is not a unique scheme that engenders either supportive or maladaptive effects in specific cerebral areas [9]. Alternative frameworks have shown distributed, network-based mechanisms, highlighting the role of individual variability, lesion location, and time post-injury [10]. In particular, longitudinal changes of network dysfunctions across the acute, subacute, and chronic phases have been observed [11].

There are some doubts about the exact mechanism of action of tDCS over the cerebral cortex. The mechanisms of action underlying the modulation of neuronal activity induced by tDCS are still not well-demonstrated [12]. Several reports have suggested that tDCS influences different cerebral circuits, influencing the activity of NMDA and GABA receptors [13]. The polarity-dependent change in excitability claimed for tDCS may be indeed an oversimplification, thus leading to some uncertainty about the real efficacy of this brain stimulation tool [14]. Neurophysiological techniques, as motor evoked potentials and electroencephalography, have been claimed as valuable tools to assess the effects of tDCS on brain activity [15]. However, different studies have found conflicting results in terms of cortical excitability and clinical outcomes among patients, indicating the need for better targeting diagnosis and etiology when addressing individuals who may respond to treatment with tDCS [16].

Few data are still available regarding the effects of tDCS on brain metabolism. In addition to functional magnetic resonance imaging (fMRI), ^18^F-FDG PET (18F-fluoro-2-deoxy-D-glucose positron emission tomography) is a minimally invasive, well-established method for the evaluation of brain function. This method evaluates the regional cerebral metabolic rate for glucose (rCGMr). FDG is an analogue of glucose, and the brain uses glucose almost exclusively. Its extensive employment in neurodegenerative dementia studies provides information about neuronal loss or synapse dysfunction [17,18]. Although widely used in daily clinical practice, there is a paucity of studies that have examined the effects of tDCS on brain metabolism through CT-PET [19]. The available studies include healthy subjects, patients with psychiatric disorders, and with multiple sclerosis.

The use of ^18^F-FDG-PET may provide a more comprehensive understanding of the effects of tDCS by examining different aspects of brain function in vivo in terms of cerebral metabolism, neuroreceptor occupancy, and neurotransmitter activity. Additionally, the combination of tDCS with PET imaging may facilitate observations of the localized effects of stimulation on targeted brain areas and the analysis of the potential impact on remote or connected regions [19,20]. Despite its potential as a research topic in the field of stroke studies, research on PET–tDCS interactions remains limited.

This exploratory study aimed to examine the effects of transcranial direct current stimulation (tDCS) on cerebral glucose metabolism in post-stroke patients by using ^18^F-FDG PET imaging. By assessing the regional cerebral metabolic rate for glucose, we explored whether and how tDCS may modulate the brain energy consumption both locally and across connected networks in vivo. To the best of our knowledge, this is the first study to evaluate the live effects of tDCS on brain metabolism in a patient with stroke.

## 2. Materials and Methods

A 61-year-old male patient with a severe right nucleo-capsular hemorrhagic stroke that occurred 3 weeks prior was admitted to our Intensive Neurorehabilitation Care Unit. The subject’s medical history revealed a previous diagnosis of hypertension. At the onset of the symptoms, the patient was taken to the emergency department and underwent an encephalic computed tomography (CT) scan, which documented extensive right internal capsule hemorrhage with a midline shift of 7 mm, associated with a proportion of edema. The angiography study did not document any vascular malformations adjacent to the area of hemorrhagic infarction. The patient was evaluated by a neurosurgeon, who found him hemiplegic and unresponsive to stimuli, as indicated by a Glasgow Coma Scale score of 10/15. The patient underwent a ventriculostomy procedure, followed by a course of therapy aimed at reducing cerebral edema. The patient was deemed to be clinically and neurologically stable upon admission to the neurorehabilitation unit. A comprehensive program of motor, cognitive, and speech rehabilitation therapy was implemented, and an intensive workup was also performed to obtain weaning from the tracheostomy. The patient underwent a PET examination during tDCS brain stimulation (Brainstim, EMS, Bologna, Italy) at 6, 8, and 10 weeks from the injury. A clinical assessment was conducted using several scales.

During the 20-week inpatient rehabilitation program, the subject displayed a progressive recovery, despite several medical complications. He was troubled by frequent respiratory desaturations with pneumonia, acute cholecystitis necessitating cholecystostomy drainage, acute renal failure, and sepsis. Treatments with antipsychotics were introduced due to the presence of agitation, with progressive dosage adjustments made over weeks. At discharge, the main neurological impairments were constituted by plegic upper limb dysfunction and aphasia (see Table 1 for details of the clinical and functional assessments).

### 2.1. Clinical Assessment

Clinical assessments were performed during the hospitalization period (Table 1). We used the National Institute of Health Stroke Severity Scale (NIHSS), a standardized scale widely used to quantify neurological impairment after a stroke, measuring consciousness, vision, motor function, sensation, language, and attention [21]; the modified Rankin Scale (mRS) to assess functional independence [22]; the Motricity Index (MI) to evaluate motor impairment of upper and lower limbs in terms of movement [23]; and the Level of Cognitive Functioning (LCF), a scale used to assess cognitive functioning in patients with disorders of consciousness [24].

### 2.2. Transcranial Direct Current Stimulation

Anodal-tDCS. The anode (6 × 5 cm–30 cm^2^) was placed on the right primary motor area (M1) while the reference electrode was placed on the contralateral fronto-orbital area. A current of 2 mA was then applied for a total duration of 20 min. The applied current density was 0.067 (i.e., 2 mA/30 cm^2^).

Cathodal-tDCS. The cathode (6 × 5 cm–30 cm^2^) was placed on the left primary motor area (M1) while the reference electrode was placed over the contralateral fronto-orbital area. The same stimulation parameters as anodal-tDCS were applied [25].

We applied the most used protocols involving either the application of anodal tDCS to the ipsilesional hemisphere to increase the excitability of the motor cortex in the injured side or the application of cathodal tDCS to the hemisphere contralateral to the injury to decrease its excitability [26,27].

### 2.3. FDG-PET Image Acquisition, Processing, and Image Analysis

^18^F-FDG-PET scans were performed at the PET-CT on a Biograph mCT64 PET/CT scanner (Siemens Healthineers, Erlangen, Germany) in three-dimensional dynamic acquisition mode [17]. The patient fasted for a period of six hours prior to the administration of the ^18^F-FDG injection, following which a dynamic PET scan of 60 minutes was initiated immediately after a bolus injection of ^18^F-FDG (3.7 MBq/kg) via the left antecubital vein. The reconstruction of PET images was achieved through the use of an iterative time-of-flight algorithm with CT-based attenuation correction as well as scatter and random corrections. The application of motion correction during the process of image analysis was performed to minimize the occurrence of artefacts. All scans were acquired and reconstructed with the same parameters. Anodal (T1) and cathodal (T2) tDCS started 1 min prior to the injection of ^18^F-FDG and continued for the entirety of the scan. Semiquantitative metrics such as the standardized uptake value (SUV) (SUVmax and SUVmean and their relative percentage variation) were not considered, as they are less sensitive compared with voxel-based analysis, which is handled in a standardized way and is statistically aggregated. In particular, SUV measures tend to have low reproducibility, being very sensitive to small technical or biological fluctuations such as blood glucose levels, injected dose, patient weight, and uptake time [28]. Moreover, SUV calculations require operator-defined volumes (volume or region of interest). Voxel-based approaches analyze the whole volume automatically, without manual delineation, thus ensuring greater consistency among measurements on the same subject. Further quantitative measures, such as rCGMr, have not been conducted as they require arterial blood sampling during the acquisition process, which is difficult to perform on highly disabled patients.

Several PET acquisition at different timepoints and different polarities of tDCS stimulation were performed: (i) baseline cerebral ^18^F-FDG-PET (T0) performed at two weeks after admission; (ii) four weeks after admission (T1) cerebral ^18^F-FDG-PET during anodal-tDCS applied on affected hemisphere; and (iii) six weeks after admission (T2) ^18^F-FDG-PET during cathodal-tDCS applied on the unaffected hemisphere.

### 2.4. Statistics

Statistical Parametric Mapping (SPM) ver.8 was used for spatial normalization to the Montreal Neurological Institute space and spatial smoothing with a 12 mm Gaussian kernel. The mean images of the dynamic PET scan were recorded and correlated to the patient structural MRI that was previously acquired. Each processed image was compared with the recordings of 19 healthy controls.

Statistical analysis was performed using a group comparison through a 2-sample t test with the SPM contrast set to “1, −1” to identify hypermetabolism and “−1, 1” to identify hypometabolism with respect to the control group [29]. SPM global normalization is a method to normalize the total radiotracer uptake level between scans but can produce false-positive or false-negative findings, especially when the areas of metabolic changes are extensive. To avoid this problem, we used the cerebellum as a regional normalization reference. The resultant t statistics data were created with a threshold of *p* < 0.001 and a cluster extension of 120 voxels.

## 3. Results

The tDCS sessions were well-tolerated, and no serious side effects were reported during the sessions.

### 3.1. Baseline ^18^F-FDG-PET (T0)

**Figure 1:**
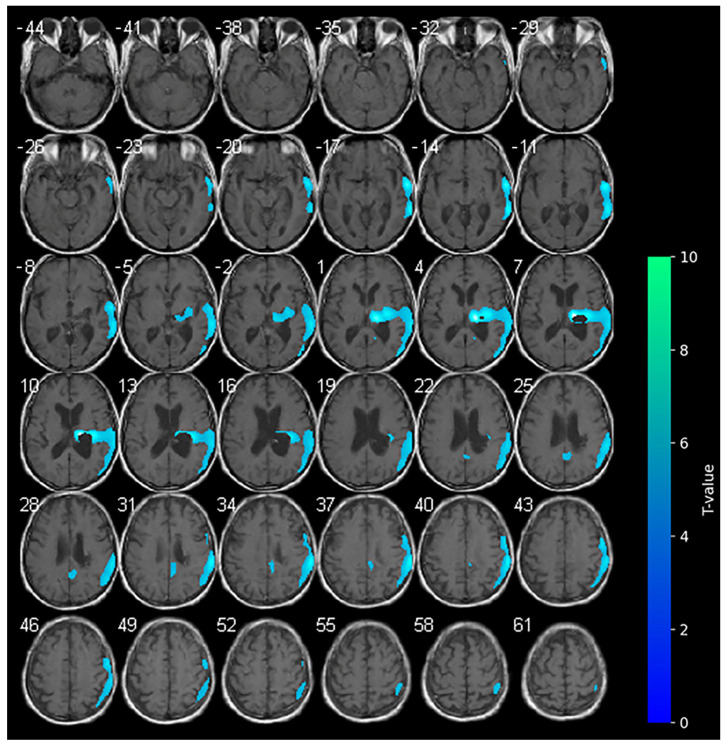
Axial slices showing the results of the SPM analysis (*t*-maps) at the baseline. The blue areas indicate the locations where the voxel values of the patient were significantly hypometabolic from the normal control group (*p* > 0.001). The brightness of the color represents the *t*-value.

At the baseline, cerebral ^18^F-FDG-PET documented marked hypometabolism in the right nucleo-capsular hemorrhagic lesion and reduced uptake of the tracer on the right side at the superior and middle temporal gyrus, supramarginal gyrus, cingulate gyrus, and precentral gyrus (Figure 1). At the baseline (T0–week 2), the clinical picture of the enrolled subject was characterized by marked neurological impairments (as indicated by an NIHSS score of 19 and an mRS score of 5), suggesting severe disability in daily activities. The patient exhibited a complete loss of motor function, as evidenced by an MI score of 0 in both upper and lower limbs. Furthermore, the subject displayed impaired consciousness, with the LCF scored at 5. While the subject demonstrated capacity to respond to simple commands, their performance was non-purposeful and fragmented when complex commands were issued. Gross attention to the environment was evident; however, the subject demonstrated an inability to concentrate on specific tasks. Verbalization was often confabulatory, and the memory was notably compromised, with limited capacity for the retention of new information. The subject’s behavior was deemed to be appropriate.

### 3.2. ^18^F-FDG-PET and Anodal-tDCS (T1)

**Figure 2:**
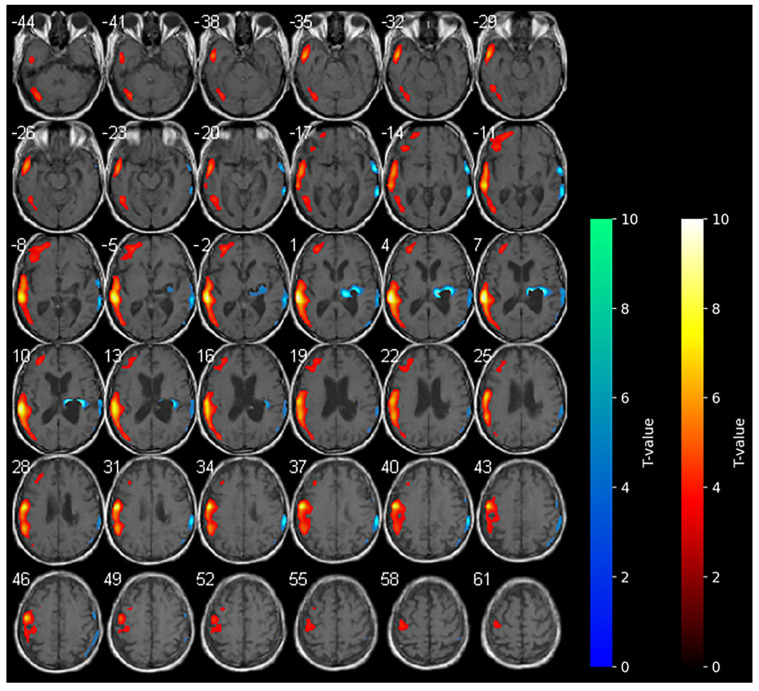
SPM analysis results (*t*-maps) during anodal-tDCS. The red and blue areas indicate voxels of significantly increased and reduced metabolism in comparison to the normal control subjects, respectively (*p* > 0.001).

Marked hypometabolism of the right hemorrhagic nucleo-capsular lesion persisted, but interestingly, a reduction in the extension of hypometabolic areas was noted compared with the baseline in the right frontal and parietal cortices. Furthermore, an increase in the uptake of the tracer was detected in the contralateral non-stimulated hemisphere (Figure 2).

During anodal tDCS, consistent with the reported data in the literature, the diffuse areas of hypometabolism observed in the affected hemisphere at the baseline were diffusively reduced, resulting in increased metabolism in these areas. In contrast with the main hypothesis of anodal tDCS functioning, the increase in metabolism in the contralateral unaffected hemisphere during anodal tDCS was unexpected.

At the second evaluation, a slight improvement was noted in cognitive functioning, as evidenced by an increase in the LCF score to 6. The responses to elementary commands demonstrated a greater degree of consistency, and the commencement of complex tasks was observed, albeit with significant delays and a certain degree of confusion. A greater degree of self-awareness was evident, accompanied by appropriate behavior. However, the subject exhibited severe impairments in recent memory and cognition, as evidenced by impaired performance in problem-solving tasks and judgment. Globally, no significant changes were observed in neurological severity or functional status as the NIHSS, mRS, and MI scores did not change.

### 3.3. ^18^F-FDG-PET and Cathodal-tDCS (T2)

**Figure 3:**
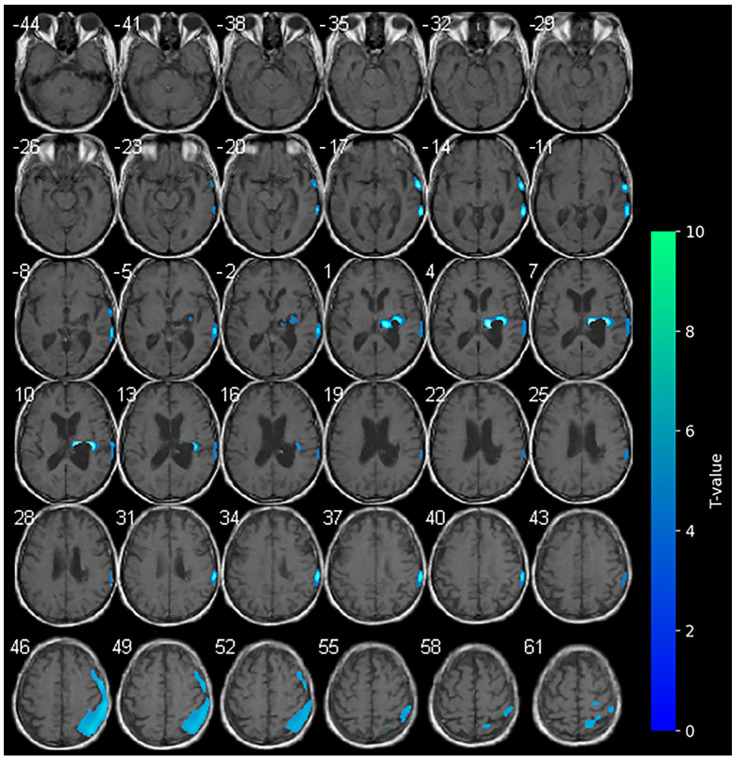
SPM analysis results (*t*-maps) at T2. The blue areas indicate voxel values of the patient that were significantly hypometabolic from the normal control group (*p* > 0.001).

No variations in the marked hypometabolism were detected in the right hemorrhagic nucleo-capsular lesion. The right fronto-parietal cortex showed reduced metabolism, a condition which was not previously observed at the baseline. No areas of significantly increased metabolism were appreciable during cathodal-tDCS (Figure 3).

A clinical improvement was observed at T2. The NIHSS score decreased to 16 and the mRS improved to 4, suggesting a partial recovery of function and reduced dependency in some basic activities of daily living. Of note, motor function showed early indications of recovery, as evidenced by an increase in the MI scores to 9 for the upper limb and 18 for the lower limb. The LCF demonstrated stability at level 6, even though the responses to requested commands were consistently more suitable.

## 4. Discussion

The use of transcranial direct current stimulation (tDCS) has gained increasing interest due to its modulatory effects on cognitive and motor functions. Alterations in synaptic function following a stroke, including reduced excitability, the formation of aberrant neural connections, and dysregulated plasticity, are hypothesized to hinder recovery. Furthermore, the affected hemisphere exhibits reduced excitability, leading to compensatory hyperexcitability in the contralateral hemisphere. This, in turn, results in a further reduction in the activity of the affected hemisphere, with a severe imbalance of the two hemispheres negatively affecting upper limb motor function [30]. These maladaptive changes are frequently reflected in aberrant brain activation patterns seen in imaging studies and the shifts in excitability observed in transcranial magnetic stimulation (TMS) studies, highlighting the need for the careful modulation of tDCS to optimize its therapeutic effects.

Despite a large number of studies, there is still conflicting consensus regarding the efficacy of tDCS in stroke rehabilitation, suggesting caution in extensive clinical application. ^18^F-FDG PET could aid in examining and describing the role of tDCS on the central nervous system in vivo. By measuring cerebral glucose metabolism, it may be able to determine the metabolic effects of tDCS and correlate regional brain metabolism with neuropsychological, behavioral, and physiological outcomes. The ‘metabolic trapping’ properties of fluorodeoxyglucose positron emission tomography can be used to gain a more detailed understanding of the mechanisms behind different tDCS applications (e.g., different intensities, durations, electrode mounts, or brain targets) [19].

The present findings appear to corroborate extant hypotheses concerning the effects of tDCS on patients with stroke [31], but concurrently provide new insights. In our study, we report unexpected brain metabolism in the contralateral hemisphere determined by anodal tDCS. According to the literature, during anodal-tDCS, the widespread areas of hypometabolism observed in the affected hemisphere at the baseline were significantly reduced (see Figure 1), indicating a global increase in the metabolism within the affected hemisphere. The increase in brain metabolism of the contralateral unaffected hemisphere during anodal-tDCS of the affected hemisphere is instead quite unexpected. It is possible that the stimulation of the affected hemisphere conversely modulates anatomical and functional networks activating the contralateral hemisphere [32,33]. The clinical improvements observed in our patient could be related to the reduction in hypometabolic areas in the affected hemisphere following anodal-tDCS, also suggesting the restoration of neuronal activity function in regions previously suppressed due to stroke-related damage. The unexpected increase in metabolism in the contralateral (unaffected) hemisphere may be indicative of a compensatory or network-level reorganization process, in which stimulation of the affected primary motor cortex influences functionally connected regions across hemispheres [11], thereby highlighting the distributed nature of neuroplastic responses to tDCS.

With regard to cathodal-tDCS administered on the unaffected side, brain metabolism appeared reduced in the ipsilateral hemisphere, as expected. In the occipital and frontal regions of the affected side, we observed hypoactivation that was not present at the baseline. The expected reduction in metabolism during cathodal stimulation may indicate a suppression of maladaptive hyperactivity in the local stimulated area, also resulting in a more balanced interhemispheric interaction. In addition, the PET images may reflect the patient’s progressive clinical improvements, including reductions in NIHSS and mRS scores and modest gains in motor function, particularly in the lower limb. Furthermore, the observed progressive improvement in cognitive performance may be linked to enhanced network integration with a more efficient brain network reconfiguration (cortical-subcortical) [34].

Concomitantly, we acknowledge the impact of spontaneous post-stroke neuroplasticity on the metabolic changes observed in our patient. Indeed, as post-stroke recovery progresses, particularly in the subacute phase, cerebral metabolism and brain activation patterns evolve over time [10,11,35,36]. However, our use of ^18^F-FDG PET imaging during the active application of tDCS, rather than in a resting state, was specifically intended to explore real-time neurophysiological responses to the stimulation protocol. While it is not possible to fully exclude the contribution of natural recovery processes, the temporal proximity between stimulation and imaging, and the metabolic differences between anodal and cathodal sessions, suggest a modulatory effect of tDCS.

In a retrospective study in patients with subacute and chronic minimally conscious states, the lack of specific behavioral patterns was observed due to the high heterogeneity and variability of brain damage present in the patients [37]. Additionally, it was noted that individuals who responded to the treatment were those who had preserved metabolic function (as measured by ^18^F-FDG-PET) and grey matter (as measured by functional MRI). In patients with severe clinical conditions, the areas metabolically preserved in responders include the left dorsolateral prefrontal cortex, the medial-prefrontal cortex, the precuneus, and the thalamus. Some authors have stated that independent of the variability of the cortical damage, residual brain activity in the stimulated area is necessary for an effective stimulation. Even if we applied this to the primary motor cortex, our findings can be considered consistent with those of Thibaut and colleagues [37], noting a reduction in the extension of hypometabolic areas and an increase in the contralateral non-stimulated hemisphere in anodal stimulation.

During tDCS intervention, functional changes were observed in interconnected brain structures, providing different insights into the recovery process, which require further examination. In such cases, ^18^F-FDG-PET has provided evidence consistent with that in the literature such as the role of the thalamus in modulating pain in patients with multiple sclerosis [38]. The modulation of the cortical excitability by tDCS in chronic pain pathophysiology has also been effectively demonstrated in studies using PET, providing insights into the involvement of multiple modulating systems and neurotransmitters including glutamate, dopamine, and endogenous opioids [39,40].

### Limitations and Perspectives of the Study

Our results should be considered with caution in light of their limitations. The study was conducted on a single subject, limiting the generalizability of the outcomes. The findings of our study should be considered exploratory and hypothesis-generating rather than confirmatory. The absence of control conditions, in both the interventions (sham-tDCS) and patient (unstimulated PET session), makes the attribution of changes to tDCS uncertain. By extending the study to other individuals in analogue protocols and by including a control group, we will be able to evaluate our outcomes in light of spontaneous recovery or as an effect of other treatments (as rehabilitation) as well as by reducing the influence of other confounding factors. In fact, the subject demonstrated a slow but progressive recovery over the six weeks, primarily in motor and global functional domains, with early cognitive improvement stabilizing by week 4. These effects can be influenced by several variables [14] including age, gender, hormones, handedness, cognitive ability, neurological or psychiatric disorders, medications, neurotransmitter levels, and differences in head anatomy. Furthermore, the mechanisms mediating the immediate effects of tDCS may differ from those responsible for longer-term changes. Therefore, all potential sources of variation and the associated risks and benefits should be taken into consideration during the design and execution of tDCS studies with PET.

In this work, although metrics such as SUVmax and SUVmean are easier to interpret and understand, we excluded them because SUV measures tend to have low reproducibility, being very sensitive to technical or biological fluctuations compared with voxel-based methods. In larger cohort studies, it will be necessary to also verify the semiquantitative measures to confirm our results and compare with data present in the literature. Then, we selected a *p* < 0.001 for the quantitative measures of PET acquisition. This is more restrictive than the commonly used *p* < 0.05 threshold. Our results could also be limited, with the probability of spurious findings being non-negligible, as the risk of false positives increases when no correction for multiple comparisons is applied.

It is essential that future studies, also including repeated unstimulated baselines, will be conducted in order to discriminate the stimulation-specific effects from intrinsic recovery neuroplasticity. Nevertheless, these outcomes are noteworthy since they demonstrate the immediate impact of a tDCS session on brain metabolism (both anodal and cathodal). No analogue study has been conducted on subjects with sABI caused by stroke.

Considering the observed metabolic changes, it is hypothesized that these findings may offer preliminary insights that could inform the optimization of future rehabilitation strategies. Specifically, the modulation of cerebral glucose metabolism in both hemispheres suggests that tDCS protocols might benefit from approaches that not only consider the site of the lesion, but also the functional state of connected networks. These results encourage further investigation into how real-time metabolic responses to stimulation could serve as biomarkers to tailor and adjust tDCS parameters in a patient-specific manner, potentially enhancing therapeutic efficacy in neurorehabilitation.

These results should be considered for the comprehension of the analysis of brain metabolism during tDCS stimulation.

## 5. Conclusions

The organization of the brain after stroke is a process that varies among patients. This study shows that ^18^F-FDG-PET is a tool that is able to reveal the tDCS mechanism of action in vivo. However, PET and tDCS studies are currently in an exploratory stage, and more studies with larger sample sizes are needed. The preliminary findings of this study indeed underline the need for future imaging studies combining tDCS with sensory stimulation in a large sample to facilitate clinician decision-making.

## Figures and Tables

**Table 1:** Clinical and functional assessment at three time points and at discharge.

Neurological and Functional Severity Scales	T0–Week 2	T1–Week 4	T2–Week 6	T Discharge–Week 20
NIH Stroke Scale (range 0–42)	19	19	16	7
Modified Rankin Scale (range 0–6)	5	5	4	4
Motricity Index Upper Limb (range 0–100)	0	0	9	9
Motricity Index Lower Limb (range 0–100)	0	0	18	28
Level of Cognitive Functioning (range 1–8)	5	6	6	7

## Data Availability

The process has been summarized into the text and all the images of interest are present in the manuscript.

## Abbreviations

The following abbreviations are used in this manuscript:

tDCS	Transcranial direct current stimulation
rCGMr	Regional cerebral glucose metabolic rate
CT	Computed tomography
^18^F-FDG PET	18F-Fluoro-2-deoxy-D-glucose positron emission tomography
NIHSS	National Institute of Health Stroke Severity Scale
mRS	modified Rankin scale
MI	Motricity Index
LCF	Level of Cognitive Functioning
SPM	Statistical Parametric Mapping
